# Dental Items

**Published:** 1871-07

**Authors:** 


### EDITORIAL DEPARTMENT.

---

*Dental Items.*—In a recent clinical lecture on "Malignant Diseases of the Jaws," delivered at the University of Pennsylvania, and reported for the *Medical and Surgical Reporter*, Dr. J. E. Garretson spoke as follows:

Malignant growths will frequently present themselves to your notice in this as in other parts of the body, and it will sometimes be a difficult question for you to decide whether life will be prolonged or comfort enhanced by an operation. Study your cases, gentlemen ; obtain all the knowledge from books, teachers, clinics, and all other sources, but learn to be independent thinkers —learn to use the faculties with which God has endowed you, then you will become truly useful, and will yourself recognize it. Because one scirrhous breast is removed—because one cancerous jaw is resected, do not conclude that all are to be similarly treated. While you are physicians be also philosophers.

In cases of epithelioma there can seldom be a doubt as to the course to be pursued when the disease is seen in its early stages; and here let me advise you never to allow your patients to delay such a procedure for a month, or even a week, if circumstances are favorable, for it is not within our knowledge to say the moment at which constitutional contamination is to occur. Numerous cases have come under my observation where persons have lost their lives simply by neglect of this injunction.

In all resections of the jaw be thorough in the removal of all parts, even well outside the disease; yet at the same time pay attention to the after comfort and appearance of your patient. For instance: a young lady came to my office, some years since, with an epulo-fibrous tumor, occupying the lower jaw, which I immediately saw was of the recurrent type. Wishing, however, to spare all needless deformity, I advised her to have simply the alveolar border resected, with the distinct understanding however, that there might be a return of the difficulty. In the event of such return I showed her that a second operation could be performed which would still preserve the contour of her features, by leaving simply the rim of bone along the base of the jaw, and should further trouble arise, a complete section could then be made. Several surgeons had advised complete removal at once, but I felt justified in the course advised, by the fact that life was not in imminent danger, and the major operation was at any moment available.

I performed the first section and it was not a success, the disease soon reappearing, and again I was most strongly urged to make a complete resection ; yet I remained firm to my first convictions of right, and performed the second, leaving a complete rim of bone along the base, and this time I was gratified to find that I had not only removed the disease (there having since been no tendency to return), but had also saved her attractive face. Spare the features then, when possible, but do not do so when it interferes in any way with safety.

Simple and compound cysts, osseous tumors and exostoses may all become causes which will compel you to remove portions of this bone.

These osteomata are not common ; but when situated upon so prominent a portion of the body will demand relief. Upon the teeth themselves we sometimes find true ivory exostoses. But these are quite rare, and will but seldom demand removal of more than a small portion of the adjoining alveolar process. These ivory exostoses, consisting of compact bony tissue, with Haversian canals and true lamellar systems, may however, develop in the bone itself.

Giant-celled sarcomata are also found in the lower jaw, but their removal is of doubtful propriety.

Cartilaginous tumors, when invading the jaw, usually affect the bone in its entire extent, and are rapid in their growth, tending to quick destruction of all the surounding parts, although Lebert mentions a case (*Abhandlungen*, p. 197) where Diefenbach removed such a tumor by three operations and the patient recovered.

I have now to show you a patient who presents herself with a small tumor upon the right alveolar process of the lower jaw, at the canine tooth, which is variable in its size, being sometimes large and dense, while again it is soft and flaccid. This I can only attribute to its being an erectile growth, analogons to the nævi, and such it truly is—an epulo-erectile tumor. I believe it to be associated with the periosteum, and my probe reveals the fact that the underlying bone is also diseased. I shall, therefore, resect this and the adjoining alveolar process, using the cutting forceps to accomplish it, after first dissecting off the tissues of the gum.

Here is a woman who was before you last winter, suffering from necrosis of the jaw, consequent upon neglected alveolar abscess. As I then told you, we had little to do but to support the system and endeavor to assist nature in the separation of the bone slough. This, you will remember, we proposed to do by the injection of stimulating materials, such as equal parts of tinct. iodine and tinct. capsic. comp., and afterward by an injection of acid. sulph., one part to seven of water, as recommended by Pollock and others. In regard to this sulphuric acid treatment, although you have seen me use it before the class, yet I am undecided as to its utility. I do not condemn it, neither can I heartily give it my support, since any remedy in such a slow operation as is the separation or decomposition of a sequestrum, should be tried before its true merits or demerits may be recognized. Nature does so much herself that we must be guarded in our statements as to the assistance which we render. Because one case recovers or does well under a certain form of treatment proves nothing—it is only as the testimony of one witness needeth further corroboration.

To the above measures we have added tonics, good food and stimulants, as well as the attempt to save such portions of the bone as might be partially dead, by stripping off the periosteum in advance of the disease by means of little tents of cotton or sponge, thus saving the osteo-genic powers, but all these have been unsuccessful.

You have become familiar with all the different steps of the treatment, as she has been repeatedly before you, and you will remember that she has never presented that robust, healthy appearance which would have been favorable to an arrest of this death of the bone. Some two months ago you will remember that I found a large sequestrum lying in the tissues, which was removed by an internal incision, and proved to be the entire ramus, save the condyloid processes. That this was not all the necrosed bone is proven by the characteristic pouting teat-like prominences which you see at the orifices of these pus discharging sinuses, and this same fact is also made evident by the probe. The entire remaining portion of that side (the right) is dead, and is now only a foreign body, increasing drain and enfeebling the patient.

In regard to resection of this bone for necrosis you have heard me express my views, especially in regard to phosphor-necrosis, since the bone is often in that porous, spongy condition that the remaining portion easily absorbs deleterious materials, and pyemia is the fatal termination of many cases. In the present case, however, the ramus is already taken away, and the only remaining portion is that from the angle to the symphysis, so that the above danger is therefore lessened.

I have been hoping to save some portion of this jaw, but now find it impossible, and propose to-day to remove all the portion alluded to. This will make a resection of considerable magnitude, and will necessitate, because of peculiarities in this case, an external incision from the centre of the lower lip to the point of the chin, and thence along beneath the bone of the jaw as far as the angle; I am afraid to risk the cul de sac which would result from an internal operation. In regard to external incisions upon the face, I would advise you, gentlemen, to spare the resulting prominent marks which must necessarily be permanent, provided you can do it without interference with the success of the operation and safety of your patient. The use of a mouth stretcher will render it possible to perform many resections without any division of the skin, but when the section is large I would not oppose you in exposing the parts, particularly in cases like this one, when pus-poisoning is the great danger to be apprehended. If you are careful in the approximation and adjustment of your wound, a union may be secured so rapid and perfect that the resulting scar will be insignificant. Safety and freedom, must be your first guide ; appearance the second.

[The operation was then commenced by making the incision as above described, and turning back the flap so that a free exposure was obtained. The soft parts were now freely dissected and a saw carried through the symphysis, when a strong pair of forceps lifted it easily from its bed without further labor, since the angle and ramus were already absent from the previous operation. The facial artery was not divided, and the only vessel requiring ligatures was the inferior labial. The parts were approximated most accurately, and the alum water dressings applied. There were no unfavorable symptoms, and the part healed most kindly, both externally and internally, so that in two weeks all seemed well, and there was no discharge in either direction.—De F. W.]

At a recent meeting of the Pennsylvania Association of Dental Surgeons, Prof. Wildman spoke as follows on the fusible alloys:

I consider that Weston's metal is not pure tin, as it makes a sharper impression than can be made with pure tin. I have seen cases when, after being exposed to the air for a long time, Weston's metal becomes tarnished far more than pure tin would be, I suppose it contains antimony. Tin is an old substitute for gold or rubber as Dr. Haden used it in 1820. He said:—I show you a case with a silver base, with the blocks attached by casting tin around them. This case has been worn over six years. Tin when placed in direct contact with gold and silver, becomes changed in character and if rendered brittle after having been worn in the mouth. This, he attributed to galvanic action. He had made many temporary sets on silver base, with blocks fast tened by tin, but had never secured blocks to a gold plate but once. Another case was worn fourteen years, and did good service until the latter part of the time. He could not recommend it for so-called permanent sets. He considered this an exceptional case. In making this kind of work, or that of the fusible alloys, the plaster composing the mould should contain a large proportion of silex, feldspar or asbestos, to render it more porous and prevent the mould from cracking by the heat to which it would be subjected. Vent holes should be made leading from the most elevated part of the mould to allow a free egress of air. The mould, at the time of pouring, should also be heated nearly or to the fusing point of the metal cast into it, to prevent its being chilled too soon. I have never made a case of the fusible alloys, as I have no confidence in them. I have more confidence in block tin. I think none of the fusible metals will resist the action of the acids of the mouth as well as pure blook tin. Dr. Blandy's metal is made of tin, 10 parts; bismuth, 1 part; antimony, 1 part; silver, one part.

A method of taking "impressions," described by Dr. B. F. Rosson, at a recent meeting of the Mad River Dental Society, is reported in the *Dental Register*, as follows:

The principle consists in obtaining an impression only of the parts upon which the plate is to rest; this is done by using for the lower jaw, a very narrow impression cup, and almost flat instead of deeply concave, as they usually are. This may be bent so as to partake as nearly as possible the form of the ridge of the jaw. A sheet of softened wax is then put upon the cup or holder, and then pressed upon the jaw until a good impression is secured; then trim down the wax just to the size the plate is to be, now use this for the plaster impression cup; a very small portion of plaster properly prepared will be sufficient to obtain a very perfect impression. The plaster should at no point be permitted to pass much beyond the proposed border of the plate, this to prevent pressing and distending the soft tissues on both sides of the ridges, such distension usually draws the mucous membrane of the gum out of position. Immediately after the cup with the plaster is placed on the jaw, the tongue should be raised above the cup and kept there till the plaster has set.

The cup for the upper jaw should not extend much beyond the ridge all round, so that the same principle is observed as described for the lower.

